# Fulminant Bilateral Streptococcus pneumoniae Endophthalmitis in a Kidney Transplant Patient With Prior Herpetic Keratopathy

**DOI:** 10.7759/cureus.100898

**Published:** 2026-01-06

**Authors:** Matheus Mota

**Affiliations:** 1 Internal Medicine, Universidade Federal de São Paulo, São Paulo, BRA

**Keywords:** endophthalmitis, herpetic keratopathy, immunosuppression, • kidney transplantation, streptococcus pneumoniae

## Abstract

Streptococcus pneumoniae endophthalmitis is an uncommon but highly aggressive intraocular infection, particularly in immunocompromised patients. Rapid clinical deterioration with irreversible visual loss and systemic complications is frequent. This report describes a fatal case of bilateral pneumococcal endophthalmitis in a kidney transplant recipient with chronic herpetic keratopathy.

A 58-year-old woman with stable renal graft function and a history of recurrent herpetic keratitis underwent left corneal transplantation. Fifty days postoperatively, she presented with worsening ocular pain and visual loss. Microbiological cultures obtained from corneal scrapings and vitreous aspirate revealed *Streptococcus pneumonia*. Intravenous and intravitreal broad-spectrum antibiotics were initiated, and retransplantation of the left cornea was performed. Despite timely intervention, the patient developed acute kidney injury, suspected herpetic meningoencephalitis, and septic shock, ultimately progressing to multiorgan failure and death.

This case highlights the fulminant nature of pneumococcal endophthalmitis in immunosuppressed patients and underscores the importance of early microbiologic diagnosis, multidisciplinary management, and tailored immunosuppressive adjustment.

## Introduction

Endophthalmitis is a severe intraocular infection that can result in irreversible vision loss if not recognized and treated promptly. Although postoperative etiologies account for the majority of cases, immunocompromised patients are also susceptible to endogenous dissemination and accelerated disease progression. *Streptococcus pneumoniae* is an infrequently isolated pathogen in large endophthalmitis case series, accounting for only a small proportion of reported cases when compared with more common organisms such as coagulase-negative staphylococci and Gram-negative bacilli. Despite its relative rarity, pneumococcal endophthalmitis is consistently associated with a fulminant clinical course, marked intraocular inflammation, rapid ocular destruction, and poor visual prognosis [1-5].

Solid organ transplant recipients represent a vulnerable population due to chronic immunosuppression, impaired host defense mechanisms, and increased susceptibility to both opportunistic infections and surgical complications. Patients with chronic herpetic keratopathy may also exhibit compromised epithelial integrity, prolonged exposure to antiviral therapy, and delayed inflammatory responses, further increasing vulnerability to secondary bacterial infections [5].

This report describes a case of fulminant bilateral pneumococcal endophthalmitis in a kidney transplant recipient following corneal transplantation, underscoring the diagnostic challenges, systemic deterioration, and the need for rapid intervention.

## Case presentation

A 58-year-old woman with end-stage kidney disease had undergone deceased-donor renal transplantation 15 years earlier, with stable graft function and baseline serum creatinine levels ranging from 1.5 to 2.0 mg/dL. The patient’s long-term immunosuppressive regimen included tacrolimus 2 mg twice daily. Mycophenolate mofetil had been discontinued two years earlier because of severe ocular viral disease. Relevant comorbidities included atrial fibrillation, osteoporosis, and heart failure with preserved ejection fraction.

In November 2024, the patient underwent left penetrating keratoplasty due to chronic herpetic keratitis with progressive visual loss and structural corneal compromise despite optimized medical therapy. Approximately 50 days postoperatively, she presented to the emergency department with severe ocular pain and rapidly worsening visual acuity.

Ophthalmologic examination revealed conjunctival hyperemia, an epithelial defect, and a 4.5 × 4.5 mm stromal infiltrate in the right eye. The left eye showed marked hyperemia, corneal ulceration, and an opaque anterior chamber, raising concern for severe intraocular infection. Empirical treatment was initiated with topical moxifloxacin and oral acyclovir. Oral doxycycline was added as adjunctive therapy due to its anti-inflammatory and anti-collagenase properties, aiming to reduce corneal stromal degradation in the setting of severe keratitis and recent corneal transplantation. Corneal scrapings and vitreous aspirate were obtained for microbiological analysis.

An urgent repeat keratoplasty of the left eye was planned but was briefly postponed because of atrial fibrillation with rapid ventricular response, requiring cardiovascular stabilization. There was no clear evidence that this short delay significantly influenced the clinical outcome. After stabilization, repeat keratoplasty was successfully performed.

Microbiological cultures from both corneal and vitreous samples grew Streptococcus pneumoniae, which was susceptible to ceftriaxone, levofloxacin, moxifloxacin, and vancomycin, and resistant to erythromycin and trimethoprim-sulfamethoxazole. Minimum inhibitory concentration values were unavailable, and antimicrobial susceptibility was reported according to standard clinical breakpoints. Intravenous ceftazidime and vancomycin were initiated, along with intravitreal administration of both agents. After microbiological confirmation, antimicrobial therapy was de-escalated to intravenous ceftriaxone at a dose of 2 g every 12 hours, with a planned treatment duration of four to six weeks. Intravenous acyclovir was continued owing to concern for concomitant viral reactivation.

Within 48 hours of targeted antimicrobial therapy and surgical intervention, the patient developed rapid systemic deterioration, including acute kidney injury with serum creatinine rising from 1.8 to 3.89 mg/dL (KDIGO stage 3), leukocytosis, thrombocytopenia, and markedly elevated inflammatory markers (Table 1). Tacrolimus was discontinued, and prednisone (20 mg/day) was initiated. Over the following 24 hours, she progressed to hemodynamic instability and altered mental status, prompting transfer to the intensive care unit. The patient subsequently developed septic shock, necessitating mechanical ventilation, vasopressor support, and renal replacement therapy.

**Table 1:** Laboratory Results WBC: white blood cell count; CRP: C-reactive protein; CSF: cerebrospinal fluid.

Test	Result	Units	Reference Range
Serum creatinine	3.89	mg/dL	0.6–1.2
Urea nitrogen	121	mg/dL	7–20
WBC count	18,700	/µL	4,000–11,000
Neutrophils	89	%	40–70
Platelets	1,42,000	/µL	150,000–400,000
CRP	29.8	mg/dL	<0.5
CSF glucose	50	mg/dL	45–80
CSF protein	130	mg/dL	15–45
CSF lactate	16	mmol/L	<2.8

**Figure 1:**
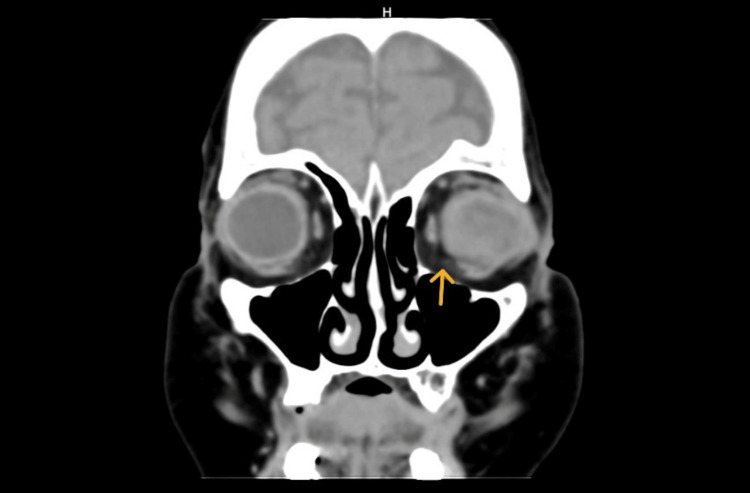
Coronal computed tomography showing rupture of the left globe (orange arrow).

**Figure 2:**
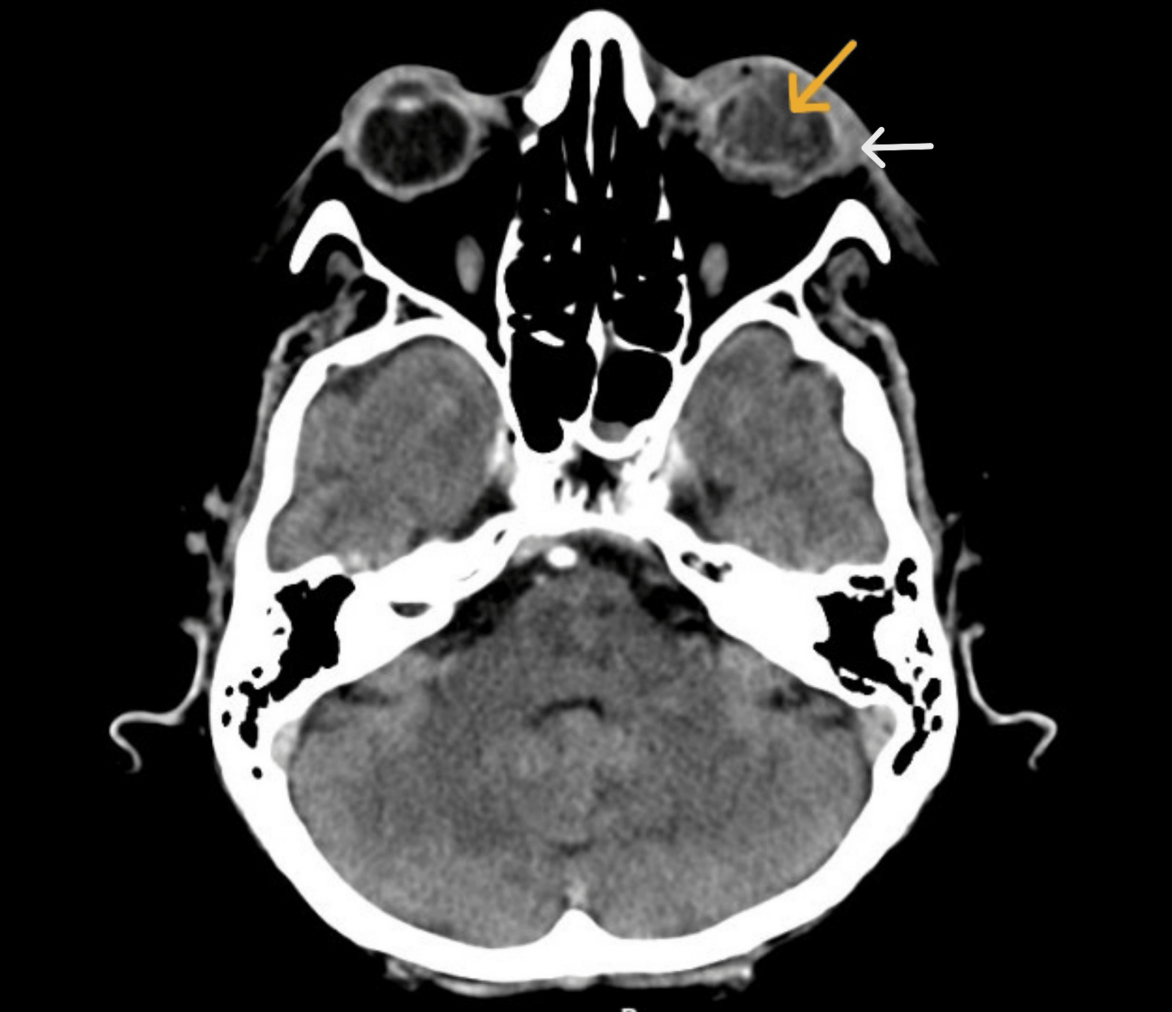
Axial computed tomography showing thickening of the left globe wall (white arrow) and heterogeneous vitreous content (orange arrow).

Orbital computed tomography demonstrated rupture of the left globe with associated intraocular and periocular inflammatory changes (Figures 1, 2). Although cerebrospinal fluid cultures and PCR testing were negative, cerebrospinal fluid abnormalities and electroencephalographic findings raised concern for viral meningoencephalitis. As magnetic resonance imaging was unavailable, empirical intravenous acyclovir was maintained in accordance with international management guidelines.

Despite maximal medical, surgical, and supportive therapy, the patient developed refractory septic shock and died from multiorgan failure.

## Discussion

Streptococcus pneumoniae is a highly virulent cause of postoperative and endogenous endophthalmitis, often resulting in severe intraocular inflammation, rapid ocular destruction, and poor visual outcomes [4-5]. Large cohort studies have shown that, although relatively uncommon compared with other bacterial pathogens, pneumococcal endophthalmitis disproportionately results in irreversible vision loss and globe loss. A multicenter Japanese cohort identified *S. pneumoniae* among the pathogens most strongly associated with severe visual sequelae [3].

In the present case, multiple predisposing factors likely contributed to the fulminant course. Chronic immunosuppression following kidney transplantation compromises innate and adaptive immune responses, increasing susceptibility to severe infections and impairing local inflammatory containment [6]. In addition, long-standing herpetic keratopathy is known to disrupt corneal epithelial integrity, alter local immune surveillance, and predispose to secondary bacterial infection. In immunosuppressed individuals, herpetic keratitis can present with attenuated clinical symptoms despite substantial structural involvement, which may delay recognition of superimposed infection and contribute to poorer outcomes [7].

The indication for penetrating keratoplasty in this patient warrants careful consideration. Corneal transplantation in the setting of chronic herpetic keratopathy and systemic immunosuppression is associated with a higher risk of graft failure and infectious complications. In the present case, the procedure was performed for progressive visual loss and structural corneal compromise refractory to medical therapy, with the aim of restoring ocular integrity while recognizing the increased risk of infection. This highlights the difficult balance between potential visual rehabilitation and the inherent vulnerability of the ocular surface in immunocompromised hosts.

Similarly, undertaking repeat keratoplasty in the setting of evolving pneumococcal endophthalmitis posed a significant surgical challenge. The primary objectives at that stage were tectonic stabilization and infectious source control rather than visual recovery. Despite prompt microbiological diagnosis and early initiation of intravitreal and systemic antimicrobial therapy, the infection progressed aggressively, culminating in globe rupture within a short time frame. This aggressive progression underscores the exceptional virulence of* S. pneumoniae* and suggests that, in severely immunocompromised patients, disease advancement may occur despite timely and appropriate interventions, leaving a very limited therapeutic window for altering outcomes.

The fulminant ocular infection was closely followed by systemic deterioration, including septic shock and severe acute kidney injury. Acute kidney injury is a well-recognized complication in solid organ transplant recipients facing systemic infection, nephrotoxic agents, and hemodynamic instability, and it is strongly associated with increased mortality. The suspected concomitant viral meningoencephalitis further complicated the clinical course. Although cerebrospinal fluid cultures and PCR testing were negative, marked cerebrospinal fluid abnormalities and electroencephalographic findings supported continued empirical antiviral therapy, in accordance with international management guidelines for suspected viral encephalitis [8-9].

Beyond its devastating ocular consequences, this case demonstrates that postoperative ocular infection in immunosuppressed patients may precipitate rapid systemic deterioration. In kidney transplant recipients, corneal transplantation performed in the setting of chronic immunosuppression and pre-existing herpetic keratopathy represents not only a localized surgical risk but also a potential portal for invasive infection with life-threatening systemic consequences. Despite early microbiological diagnosis, prompt intravitreal and systemic antimicrobial therapy, and surgical intervention, fulminant pneumococcal endophthalmitis progressed relentlessly, culminating in septic shock, multiorgan failure, and death. These findings emphasize that postoperative endophthalmitis in solid organ transplant recipients should be regarded as a systemic medical emergency rather than a purely ophthalmic complication, warranting early multidisciplinary involvement and close postoperative surveillance [10].

This report has inherent limitations. The exact source of infection could not be definitively established, and both postoperative and endogenous origins remain possible. Minimum inhibitory concentration data were unavailable, limiting more precise antimicrobial pharmacodynamic analysis. In addition, the diagnosis of viral meningoencephalitis remained presumptive, as cerebrospinal fluid cultures and PCR testing were negative despite supportive cerebrospinal fluid abnormalities and electroencephalographic findings. These uncertainties reflect real-world clinical challenges in the management of critically ill immunosuppressed patients.

## Conclusions

In immunosuppressed patients, particularly those with pre-existing herpetic keratopathy, corneal transplantation carries not only a high risk of severe ocular complications but also substantial systemic consequences. In this case, postoperative pneumococcal endophthalmitis progressed rapidly despite early diagnosis and aggressive antimicrobial and antiviral therapy, evolving from a localized ocular infection to fatal systemic deterioration. This report underscores the importance of carefully considering the systemic implications of corneal transplantation in solid organ transplant recipients, ensuring close postoperative surveillance, and involving multidisciplinary teams early when ocular infection is suspected.
